# Incidence of scoliosis in cerebral palsy: A population-based study of 962 young individuals

**DOI:** 10.1080/17453674.2018.1450091

**Published:** 2018-03-14

**Authors:** Gunnar Hägglund, Katina Pettersson, Tomasz Czuba, Måns Persson-Bunke, Elisabet Rodby-Bousquet

**Affiliations:** 1 Lund University, Department of Clinical Sciences, Lund, Orthopedics;; 2 Department of Orthopaedics, Skane University Hospital, Lund;; 3 Centre for Clinical Research, Uppsala University, Region Västmanland, Västerås;; 4 National Competence Center for Quality Registers, University Hospital, Lund, Sweden

## Abstract

**Background and purpose — Surveillance of scoliosis in individuals with cerebral palsy (CP) is important for ensuring timely diagnosis and identification of curve progression. We analyzed the incidence of scoliosis in relation to age, sex, and gross motor function in a population-based cohort of individuals with CP.**

**Patients and methods — This was a prospective register study of all 1,025 individuals born 1990–2012 in southern Sweden (1.4 million inhabitants) in the Swedish surveillance program for CP, which included >95% of the total population of people with CP in the area. Annual clinical examinations and radiographic measurement of the Cobb angle of those with a moderate or severe scoliosis were registered. We determined the incidence of scoliosis related to age, sex, and the Gross Motor Function Classification System (GMFCS) level.**

**Results — The inclusion criteria were fulfilled by 962 individuals. The number of people (140/962) with scoliosis increased up to 20–25 years of age. The incidence of scoliosis was related to age and GMFCS level. In individuals at the lowest level of gross motor function (GMFCS V) scoliosis was seen in 10/131 before 5 years of age and at the age of 20 years 75% of these individuals had a Cobb angle ≥40°. No one in the highest level of motor function (GMFCS I) developed a Cobb angle ≥40°**

**Interpretation — Surveillance programs for scoliosis in CP should be based on age and GMFCS level and should be initiated at a young age and continued into adulthood.**

Scoliosis is common in non-ambulatory individuals with cerebral palsy (CP) (Saito et al. 1998, Koop 2009). Spasticity, muscle weakness, and incomplete muscle control contribute to impaired trunk control and the development of spinal deformity (Imrie and Yaszay 2010, Tsirikos 2010). Severe scoliosis may cause additional motor dysfunction, sitting and transfer problems, compromised pulmonary function, and pain with reduced quality of life (Kotwicki and Jozwiak 2008, Fender and Baker 2011).

The reported incidence of scoliosis in people with CP varies because studies have used different definitions of scoliosis, age groups, and distribution of gross motor function. Most studies report an incidence of 20–25% (Balmer and MacEwen 1970, Persson-Bunke et al. 2012). The risk is higher in people with total body involvement, for whom an incidence of 64% has been reported (Madigan and Wallace 1981).

The overall goal for management of a spinal deformity in patients with CP is to maintain or improve functional abilities, seating, positioning, and quality of life. Close surveillance and evaluation are key components for identifying curve progression and improving or maintaining overall function. This can be done through seating adaptations (Holmes et al. 2003), spinal orthoses (Terjesen et al. 2000, Rutz and Brunner 2008) and surgical treatment (Toovey et al. 2017).

Increased knowledge of the incidence of scoliosis in an unselected group of people with CP is of value for predicting future risk of scoliosis, and identifying critical ages for surveillance.

CPUP has been a Swedish CP surveillance program for over 2 decades, and was designated as a National Quality Registry in 2005. The registry has a coverage rate of over 95%, and thereby represents almost all children with CP in Sweden (Westbom et al. 2007). CPUP aims to prevent hip dislocations, contractures, and deformities in children with CP (Hägglund et al. 2014, Alriksson-Schmidt et al. 2017).

In 2012 an analysis of the incidence of scoliosis in southern Sweden based on data from 1995 through 2008 was presented, and represented 666 children aged 0–18 years (Persson-Bunke et al. 2012). The aim of this study was to further analyze the incidence and prevalence of scoliosis related to gross motor function, sex, and age in the same area with the cohort expanded with longitudinal data between 2008 and 2016.

## Patients and methods

In CPUP, children are enrolled at the earliest suspicion of CP, and the CP diagnosis is determined by a neuropediatrician after the age of 4 years. CPUP includes a continuous standardized follow-up of gross motor function, hand function, clinical findings, and treatment. The children are examined by their local physiotherapist following a standardized method twice a year until 6 years of age and then once a year (Alriksson-Schmidt et al. 2017). In CPUP, children with CP are also followed regularly into adulthood.

Gross motor function is classified by the child’s physiotherapist according to the Gross Motor Function Classification System (GMFCS), a five-level system based on self-initiated movement, where level I represents the highest level of function and level V the lowest (Palisano et al. 2008). The clinical examination includes an assessment of the spine in a sitting position, upright and with forward bending. Any spinal deviation is graded as mild, moderate or severe scoliosis according to guidelines outlined in a manual (http://www.cpup.se, Table 1). This standardized clinical spinal assessment has shown high interrater reliability, sensitivity, specificity, and criterion-related validity compared with radiographic Cobb angle measurement (Persson-Bunke et al. 2015). In this study, scoliosis graded as moderate or severe was our event of interest. In CPUP, children 8 years and younger with a fixed scoliosis and children older than 8 years with moderate or severe scoliosis at clinical examination are examined radiographically with anteroposterior and lateral views of the entire spine. The radiographic examination is done in the standing or sitting position if possible and in the supine position if not. The curve magnitude is measured as the Cobb angle. Further radiographic examinations are scheduled based on the progression of the Cobb angle, age, and GMFCS level. In children with a Cobb angle ≥40°, surgical treatment is considered (Saito et al. 1998).

**Table 1.** Classification of scoliosis on clinical examination in CPUP

No scoliosis	
Mild scoliosis	A discrete curve visible only during forward bending
Moderate	An obvious curve visible during both extended and
scoliosis	forward bending
Severe	A pronounced curve preventing the child from attain-
scoliosis	ing an upright position without external support

In this prospective cohort study, all 1,025 children and young adults with CP in the area born between January1, 1990 and December 31, 2012, and participating in CPUP were included at baseline. The study area of southern Sweden comprises 1.4 million inhabitants. 10 children who died and 5 who moved out of the area before the age of 5 years were excluded. People with CP who moved into the area after 5 years of age (n = 58) were also excluded. After 5 years of age, 44 children died (median age 13 (6–25) years) and 37 moved out of the area (median age 9 (6–24) years). They were included in the analysis during the time they were alive and lived in the area. The clinical and radiographic measurements performed from July 1, 1995, until February 3, 2017, were used for the analyses.

### Statistics

The period prevalence of children with scoliosis was calculated as the number of children with scoliosis during the study period July 1, 1995–February 3, 2017 related to the total population of children with CP in the registry during the same period. Kaplan–Meier analysis was used to identify the age at diagnosis of moderate or severe scoliosis stratified by sex and GMFCS level, and the age at diagnosis of scoliosis with a Cobb angle ≥40° stratified by GMFCS. The numbers at risk are presented in 5-year intervals. Cox regression analysis was used to compare the risk of scoliosis in different age groups, GMFCS levels, and in males and females for both criteria. The model fulfilled the proportional hazard assumption.

Chi-square tests were used to compare differences in frequency between males and females.

The analyses were performed using Stata (IC v.13, StataCorp LP, College Station. TX, USA).

## Ethics, funding, and potential conflicts of interest

The study was approved by the Medical Research Ethics Committee at Lund University (LU-433-99). The study was funded by Stiftelsen för bistånd åt rörelsehindrade i Skåne and by the Norrbacka-Eugenia Foundation. The authors declare no conflict of interest.

## Results

There were 962 individuals (557 males, 405 females) who fulfilled the inclusion criteria. The sex distribution related to GMFCS level is presented in Table 2. During the follow-up period, 140 of the 962 individuals (15%) developed moderate or severe scoliosis based on the latest clinical examination. The scoliosis was graded as moderate in 48 cases (26 males, 22 females) and severe for 92 (50 males, 42 females). Moderate or severe scoliosis was documented in 14% (76/557) of the males and in 16% (64/405) of the females. Spinal fusion was performed in 50 (54%) of the cases, all classified as having severe scoliosis; 26/50 males (52%) and 24/42 females (57%)). The mean age at surgery was 14 (6–22) years and the median preoperative Cobb angle was 72° (40°–115°). The GMFCS levels of the individuals operated were levels III (n = 2), IV (n = 15), and V (n = 53).

**Table 2.** Sex and GMFCS distribution

	GMFCS level					
Sex	I	II	III	IV	V	Total
Male	236	114	45	75	87	557
Female	157	76	50	60	62	405
Total	393	190	95	135	149	962

Radiographic examination was registered for 128 of the 140 persons with moderate or severe scoliosis. The Cobb angle was <20° in 27 cases, of which all were graded as moderate on clinical examination. In 14 cases the Cobb angle was 20°–39°. Half of them were rated as moderate and half as severe on clinical examination. In the remaining 87 cases the Cobb angle was ≥40° (median 60° (40°–100°); 80 were classified as having severe scoliosis on clinical examination. Of the 12 children not examined radiographically, 6 had moderate and 6 had severe scoliosis. Their GMFCS levels were I (n = 2), II (n = 1), III (n = 1), IV (n = 2), and V (n = 6). The children in GMFCS level V were considered to be in too poor health to receive scoliosis surgery. The reason for not being referred for radiographic examination in the 6 children in GMFCS I–IV was not reported.

**Figure 1.**
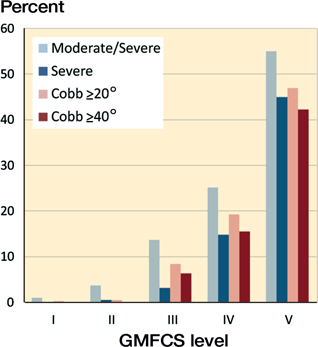
Percentage of individuals at GMFCS I–V with moderate or severe scoliosis at clinical examination and with a Cobb angle exceeding 20° or 40° at radiographic examination. Individuals with Cobb ≥40° are also included in the presentation of Cobb ≥20°.

The overall frequency and severity of scoliosis increased with GMFCS level, from 0–1% in GMFCS level I to 42–55% in GMFCS level V based on clinical or radiographic examination (Figure 1).

**Figure 2.**
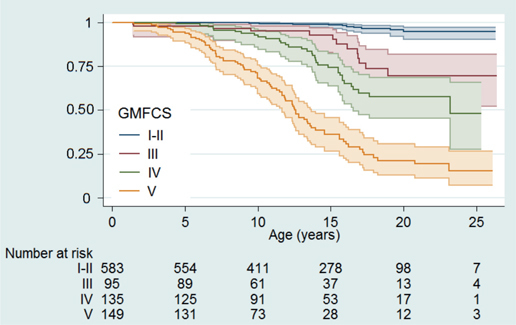
Survival function with 95% confidence interval showing the risk of having a moderate or severe scoliosis diagnosed at different GMFCS levels and ages. Numbers at risk at inclusion and at 5-year intervals reported.

**Figure 3.**
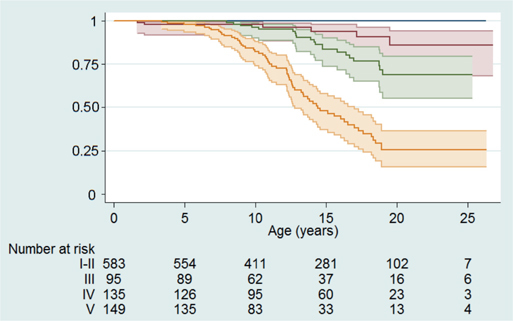
Survival function with 95% confidence interval showing the risk of having a scoliosis with Cobb angle ≥40° diagnosed at different GMFCS levels and ages. Numbers at risk at inclusion and at 5-year intervals reported. For GMFCS color codes see Figure 2.

**Figure 4.**
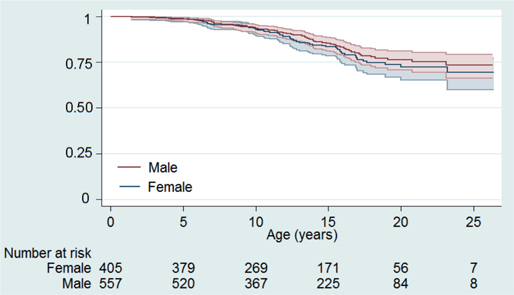
Survival function with 95% confidence interval showing the risk of having a moderate or severe scoliosis in males and females respectively. Numbers at risk at inclusion and at 5-year intervals reported.

Kaplan–Meier survival estimates based on the results of the clinical examination showed that scoliosis was seen in younger ages in children with higher GMFCS levels (Figure 2). The incidence of scoliosis increased with age and GMFCS level. At 10 years of age about 1% of the children at GMFCS I–II, 5% at GMFCS III, 10% at GMFCS IV, and 30% at GMFCS V had a moderate or severe scoliosis. At 20 years of age the corresponding percentages were 5%, 30%, 45%, and 80% respectively. Survival estimations based on the results of radiographic examination and surgery showed a similar pattern (Figure 3). At 10 years of age 2% of children at GMFCS III, 5% at GMFCS IV, and 20% at GMFCS V had a Cobb angle exceeding 40°. At 20 years of age the corresponding percentages were 8%, 35%, and 75% respectively. No child in GMFCS I–II developed scoliosis with Cobb angle ≥40°. The survival estimation based on sex showed equal development for males and females (Figure 4). In the Cox regression analysis, a high GMFCS level indicated a high risk of scoliosis (Tables 3 and 4).

**Table 3.** Cox regression analysis of the hazard ratio (HR) for developing clinically moderate or severe scoliosis in relation to GMFCS level and sex

	HR	95% CI	p-value
GMFCS level			
III vs. I/II	8	4–17	< 0.001
IV vs. I/II	15	9–30	< 0.001
V vs. I/II	53	28–100	< 0.001
Females vs. males	1.4	1–2	0.04

**Table 4.** Cox regression analysis of the hazard ratio (HR) for developing scoliosis with Cobb angle ≥40° in relation to GMFCS level ^a^ and sex

	HR	95% CI	p-value
GMFCS level			
IV vs. III	2.3	0.96–5.9	0.04
V vs. III	10	4.5–24	< 0.001
Females vs. males	1.4	0.88–2.1	0.1

* No child in GMFCS I or II had Cobb ≥40°.

## Discussion

The main findings in this study were that higher GFMCS level was a significant risk factor for the development of scoliosis, that scoliosis occurred at younger ages in individuals classified at a higher GMFCS level, and that the incidence of scoliosis continued to increase up to the age of 20–25 years. A strength of the study is that the data are based on >95% of the total population of people with CP in the area followed in a standardized way in CPUP, the Swedish national CP surveillance program.

In the Kaplan–Meyer analysis based on clinical examination first occurrence of moderate or severe scoliosis was considered an event of interest. A study of the reliability and validity of the CPUP grading system of scoliosis showed that most children with a mild scoliosis had a Cobb angle of only 5°–15° (Persson-Bunke et al. 2015). In the analysis based on radiographic examination a Cobb angle ≥40° was used as the cut-off. The rationale for this is that a Cobb angle greater than 40° has been found to predict significant progression of the magnitude of the curve, and is therefore an indication for considering surgery (Saito et al. 1998, Gu et al. 2011).

The cohort comprised 58% males, a distribution that is consistent with the overall boy/girl ratio reported for CP (Westbom et al. 2007). The proportion of males in GMFCS I–II was 60% and in levels III–V 55%. The incidence of scoliosis and the proportion operated with spinal fusion was slightly higher in females.

Scoliosis was strongly related to the child’s GMFCS level (see Figure 1). For children at GMFCS level I or II the incidence was low and similar to that of adolescent idiopathic scoliosis in typically developed children (Willner and Udén 1982). A previous study from the same area that included 666 children followed between 1999 and 2008 showed a 50% risk for moderate or severe scoliosis in children at GMFCS level IV or V at 18 years of age (Persson-Bunke et al. 2012). In that study the size of the sample did not allow separate analyses for children at GMFCS levels IV and V. The larger cohort and longer follow-up time in the present study made this separation possible and showed that the risk increased significantly from level IV to V (see Figures 2 and 3). A systematic review by Loeters et al. (2010) found 4 studies that reported a relationship between the severity of CP and scoliosis. The study by Persson-Bunke et al. (2012) showed no relationship between CP subtype and scoliosis, which is why this variable was not included in the present study.

Advances in surgical technique in recent decades have provided methods to treat scoliosis effectively while also reducing complication rates (Cloake and Gardner 2016). There are several arguments for early detection and treatment of scoliosis with increasing curve magnitude. Preoperative curve flexibility is an important predictor of the degree of curve correction obtained at surgery (Beckmann et al. 2016). A greater angle of scoliosis is associated with a higher risk of complications (Lipton et al. 1999, Master et al. 2011). Scoliosis in CP often leads to a pelvic obliquity, which can increase the risk of hip dislocation on the high side. It also results in internal hip rotation and reduced range of hip flexion on the high side (Ágústsson et al. 2017), which may result in sitting problems postoperatively.

One aim of CPUP is to prevent hip dislocation and severe contractures. Scoliosis is sometimes preceded by hip dislocation or windswept hip deformity, which can cause a pelvic obliquity and initiate scoliosis (Letts et al. 1984, Hägglund et al. 2016). CPUP has substantially reduced the prevalence of children with hip dislocation and windswept hip deformity (Hägglund et al. 2014, 2016). Consequently, the prevalence of scoliosis is probably higher than in the present study in areas without hip surveillance programs.

Scoliosis was seen at younger ages in the children at GMFCS level V. Surgery with complete spinal fusion in young children can cause problems by impairing growth of the spine and thorax, which has effects on respiratory development and function (Vitale et al. 2008). If the curve is flexible, it is possible to reduce the progression rate and delay the need for surgery by spinal bracing (Olafsson et al. 1999, Terjesen et al. 2000). Spinal brace treatment to delay surgery was rare in the study area. The exact number of children using spinal orthoses to prevent curve progression could not be obtained from the reported data. More recent techniques allow the use of “growing rods” to provide spinal support and correct deformity while allowing for growth (McElroy et al. 2012).

Not all individuals with moderate or severe scoliosis on clinical examination were examined radiographically. Some of the radiographic examinations were done in the lying position, which might have underestimated the curve magnitude in children with a flexible curve. The numbers at risk in the Kaplan–Meier analysis were low at some GMFCS levels at 20 and 25 years of age.

In summary, the incidence of scoliosis in individuals with CP was strongly related to their GMFCS level. At 20 years of age about 75% of those at GMFCS level V had a Cobb angle ≥40°. Surveillance programs should be based on age and GMFCS level and must start at young ages and continue into adulthood.

Study design: GH, KP, TC, MPB, ERB. Data collection: GH, MPB. Data analysis: GH, KP, TC, MPB, ERB. Manuscript preparation: GH, KP, TC, MPB, ERB.

*Acta* thanks Thomas Andersen and other anonymous reviewers for help with peer review of this study.
